# Gene identification and analysis of transcripts differentially regulated in fracture healing by EST sequencing in the domestic sheep

**DOI:** 10.1186/1471-2164-7-172

**Published:** 2006-07-05

**Authors:** Jochen Hecht, Heiner Kuhl, Stefan A Haas, Sebastian Bauer, Albert J Poustka, Jasmin Lienau, Hanna Schell, Asita C Stiege, Volkhard Seitz, Richard Reinhardt, Georg N Duda, Stefan Mundlos, Peter N Robinson

**Affiliations:** 1 Max Planck Institute for Molecular Genetics, Berlin, Germany; 2 Freie Universität Berlin, Fachbereich Biologie, Chemie, Pharmazie, Berlin, Germany; 3 Institute for Medical Genetics, Charité Universitätsmedizin, Berlin, Germany; 4 Max Planck Institute for Molecular Genetics, Evolution and Development Group, Berlin, Germany; 5 Center for Musculoskeletal Surgery, Charité Universitätsmedizin, Berlin, Germany

## Abstract

**Background:**

The sheep is an important model animal for testing novel fracture treatments and other medical applications. Despite these medical uses and the well known economic and cultural importance of the sheep, relatively little research has been performed into sheep genetics, and DNA sequences are available for only a small number of sheep genes.

**Results:**

In this work we have sequenced over 47 thousand expressed sequence tags (ESTs) from libraries developed from healing bone in a sheep model of fracture healing. These ESTs were clustered with the previously available 10 thousand sheep ESTs to a total of 19087 contigs with an average length of 603 nucleotides. We used the newly identified sequences to develop RT-PCR assays for 78 sheep genes and measured differential expression during the course of fracture healing between days 7 and 42 postfracture. All genes showed significant shifts at one or more time points. 23 of the genes were differentially expressed between postfracture days 7 and 10, which could reflect an important role for these genes for the initiation of osteogenesis.

**Conclusion:**

The sequences we have identified in this work are a valuable resource for future studies on musculoskeletal healing and regeneration using sheep and represent an important head-start for genomic sequencing projects for *Ovis aries*, with partial or complete sequences being made available for over 5,800 previously unsequenced sheep genes.

## Background

The accumulation of genome-wide sequence data for model organisms is of obvious benefit for many kinds of scientific analysis. Although the sheep is of great economic importance and has been used as a model organism to study such diverse topics as fracture healing [1], ovarian function [2], hypernatremic hypertension [3], and pulmonary function [4], relatively little sequence information is available for this organism, and DNA sequences are available for only a small number of sheep genes.

2030 loci, 543 of which are designated as genes, are currently listed in the ArkDB sheep database [5]. In UniGene [6] Build 7 for *Ovis aries*, a total of 5,730 EST and mRNA sequences form a total of 1714 clusters, and the EBML database [7] currently contains 10497 ESTs and 2391 mRNA entries for sheep.

Fractures, and especially osteoporotic fractures in the elderly, represent a major public health problem. As populations age, the costs of treating osteoporotic fractures, currently billions of dollars a year in the United States alone, are predicted to dramatically increase [8]. Consequently, a better understanding of the genetic processes underlying fracture healing is of great medical relevance. The process of fracture healing is a regenerative process that in many ways is similar to bone development. The fracture repair process goes through many stages, beginning with an inflammatory response and the recruitment and proliferation of undifferentiated mesenchymal cells. Mesenchymal cells from the periosteum near the fracture site differentiate into osteoblasts and induce intramembranous ossification resulting in the formation of new bone. Simultaneously, cartilage is formed at the fracture site and undergoes enchondral ossification. The newly produced bone then undergoes extensive remodeling until the original shape and structure of the bone is restored. Many of the molecular signals and processes involved in fracture healing closely resemble those involved in bone development [9-11].

In the present work, we have extracted 47 thousand ESTs from libraries developed from healing bone in a sheep model of fracture healing. We clustered our sequences together with previously identified ESTs, and identified 19087 contigs, the majority of which represented previously unsequenced sheep genes. These newly identified sequences were used to perform RT-PCR on 78 genes from the postfracture day 7, 10, 14, and 42 libraries. We confirmed differential expression for genes previously known to be involved in fracture healing and demonstrated differential expression for numerous genes not previously known to be involved in fracture healing.

## Results

### ESTs and assembly into contigs and creation of an *Ovis aries *sequence database

**Figure 1:**
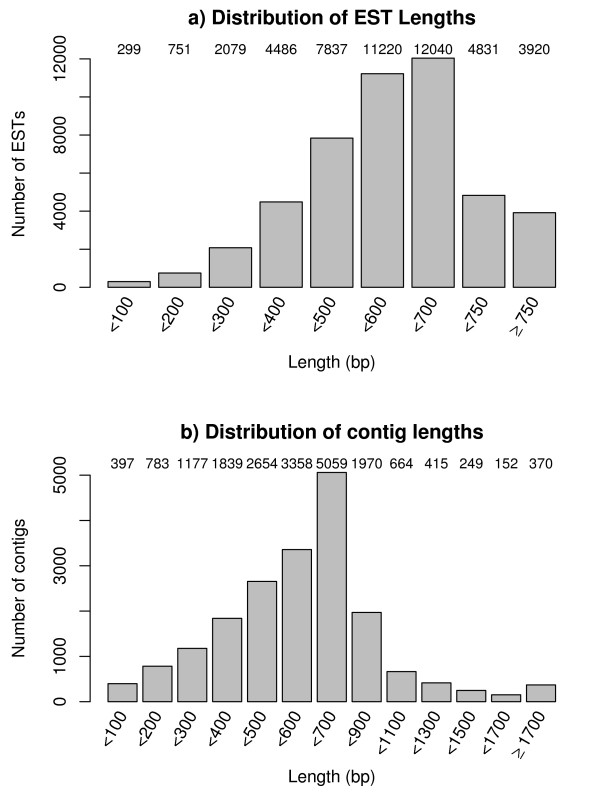
**Distribution of sequence length and EST count per contig**. a) Distribution of the read length of the ESTs after quality control, b) Distribution of sequence length of the assembled contigs.

In addition to the 47209 sheep ESTs generated from three libraries in the current project (Table 1), we extracted 10497 ESTs and 2391 mRNA entries from the EMBL database (release 82, Sept 2005). 344 sequences were rejected because less than 40 bp remained unmasked following quality control procedures. The lengths of the ESTs ranged from 40 to 928 with an average length of 555 nucleotides (Fig. 1a). All bases beyond 750 bp were masked. Using our sequences as well as the previously available sequences, 19087 contigs were assembled with an average length of 603 nucleotides (Fig. 1b). 397 contigs were shorter than 100 nucleotides and were excluded from further analysis. On average, each contig was assembled from 3.1 ESTs from all libraries, with a range of 0 to 2392 ESTs per contig (some contigs consisted only of previously available mRNA data and contained no ESTs). If the analysis is restricted to ESTs from the libraries described in this work, then each contig contained an average of 2.5 ESTs (Range: 0 – 2387). The contigs comprised of the largest number of ESTs were identified as collagen I*α*1 (2387 ESTs), collagen I*α*2 (1094 ESTs), and osteonectin (1086 ESTs). A total of 8965 contigs contained only a single EST clone.

**Figure 2:**
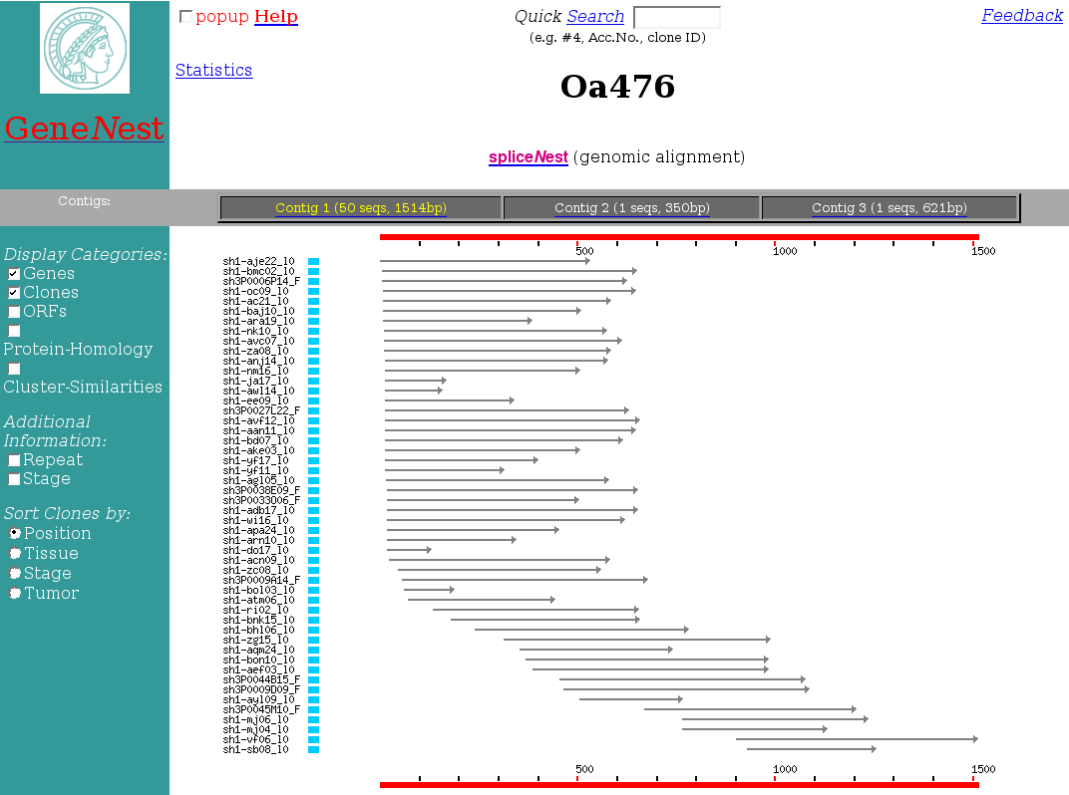
**GeneNest *Ovis aries *gene index database**. In this example, the data for integrin-binding sialoprotein (IBSP) are shown. A cluster based on three contigs, each of which can be individually displayed, corresponds to IBSP. EST sequences from which the contigs were constructed as well as their locations and libraries are displayed. A consensus sequence can be downloaded for each contig. Open-reading frames can be optionally displayed, and other options for data display are available [12]. The database is publicly available at .

We used the GeneNest package [12,13] to analyze all available mRNA and EST sequences and to present the sequences in a user-friendly internet site. The main features of the GeneNest Sheep database are summarized in Fig. 2.

### BLAST analysis of *Ovis *aries contigs to assign putative gene identities based on homology

**Figure 3:**
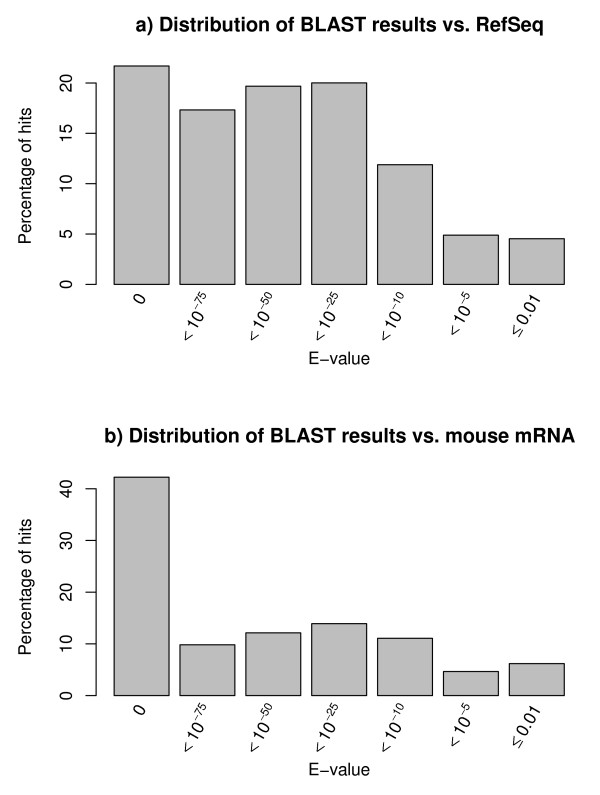
**Distribution of the e-values of different BLAST analyses**. Distribution of e-values for *Ovis aries *contigs blasted against the RefSeq protein database (a) or the nonredundant mRNA database restricted to mouse sequences (b).

BLAST searches were used to attempt to assign putative identities to the contig sequences identified in this study. Analysis against the NCBI RefSeq protein sequence database was performed, and 13149 contigs demonstrated a best e-value of 0.01 or lower (Fig. 3a). We used an arbitrary e-value cutoff of 10^-25 ^to identify significant homology to a known gene and allow a probable assignment of a sheep contig. With this cutoff, a total of 10387 contigs demonstrated significant homology to at least one other protein in the RefSeq protein database and could be assigned a putative identity, consisting of a total of 8789 distinct proteins. In cases where hits to a proteins from several species were identified, the most significant match was used to assign a putative identity. 292 contigs blasted to an *Ovis aries *RefSeq. A total of 2863 contigs (15%) had e-values of less than 10^-100 ^and are thus likely to represent true orthologs. Of the 19087 contigs, 11591 were assembled from ESTs only from the current project. 6577 were assigned to 5839 distinct RefSeq sequences by virtue of blastx e-values of 10^-25 ^or less. These sequences represent previously unidentified sheep genes. The sequences for these genes are available in FASTA format with the online supplemental material as [Additional file 1]

### Gene-Ontology analysis of the sheep fracture libraries

**Figure 4:**
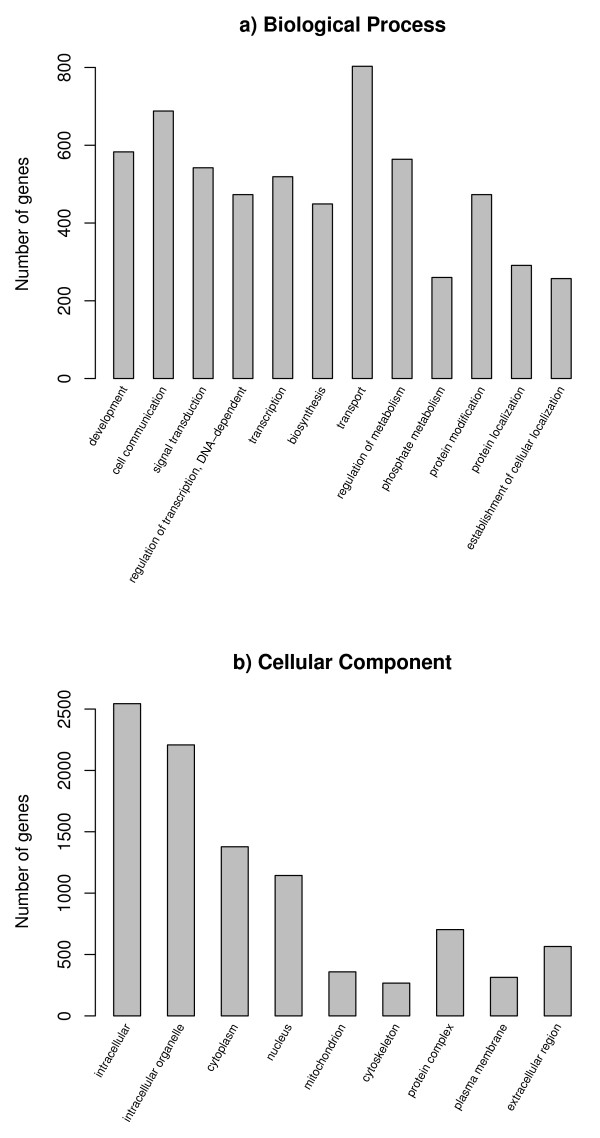
**GO analysis**. A histogram showing the counts of genes annotated to GO categories for Biological Process and Cellular Component among the 4600 mapped sheep genes for which MGI annotations of putative mouse homologs were available. Terms were selected from among the most frequently annotated and terms deemed to be particularly interesting for fracture healing. a) Terms from the subontology *biological process*. b) Terms from the subontology *cellular component*.

GO analysis was performed by assigning annotations to the sheep contigs from the MGI annotations of putative murine homologs (e-value threshold 10^-25^) in order to get an overview of the subset of genes expressed in healing fracture tissue of the sheep. Fig. 3b provides an overview of the results of this BLAST analysis. Putative homologs were found for 5637 distinct sheep genes, some of which were defined from multiple contig sequences. MGI annotations were available for 4600 of the 5637 genes. The results of the GO analysis using these annotations are summarized in Fig. 4. Many of the most frequently annotated terms in the subontology *biological process*, such as *development, signal transduction, transcription, biosynthesis, and phosphate metabolism*, reflect processes known to be involved in fracture healing and bone development. The distribution of annotations to terms in the subontology *cellular component *shows that the gene products corresponding to the ESTs identified in this work are active in many different cellular and extracellular locations.

### A comparison of the sheep fracture libraries with normal human bone libraries

During development and in related processes such as fracture healing, genes exhibit complex patterns of spatial and temporal regulation. The identification of genes that are differentially expressed during fracture healing therefore represents an important initial investigation to discover genes that are essential for fracture healing.

**Figure 5:**
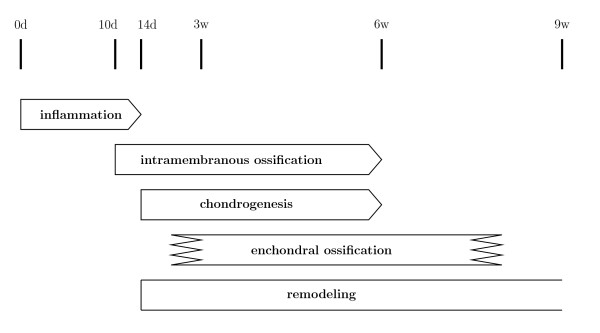
**Time course of fracture healing in *Ovis aries***. Time line showing the central processes during fracture healing in a sheep model under standardized external fixation (mechanically uncritical). The starting time and duration of each process were estimated from histological analysis at the indicated time points. The temporal extension of the enchondral ossification phase is loosely defined with the starting and ending point probably being localized between the analyzed time points (Lienau, Schell, and Duda, unpublished data).

In this work, we generated two EST libraries from early fracture healing stages (PF7 and PF10) with the goal of identifying genes important in the earliest stages of fracture healing. Following fracture, initial hemorrhage into the fractured bone is followed by hemostasis and a phase of inflammation, which lasts up to two weeks in the sheep (Fig 5). Intramembranous ossification begins around day 10. Therefore, we reasoned that genes that are overexpressed in the PF7 and PF10 libraries could represent genes with potential importance for the initiation of new bone formation. To estimate gene expression levels in the two libraries, we compared EST sequence counts using a two-tailed Fisher exact test. This analysis revealed statistically significant differential expression for only three genes; tartrate resistant acid phosphatase 5 (ACP5) increased by a fold change of 10.60 (5 to 53 ESTs), cytochrome c oxidase subunit I (COX1) increased by a fold change of 2.1 (87 to 178 ESTs), and perlecan decreased by a fold change of 3.9 (31 to 8 ESTs). We did not perform such a comparison using the third EST library, because the latter was derived from a mix of time points using PCR amplification of cDNA inserts in order to get a broader overview of the genes involved in the many stages of fracture healing in sheep.

RNA samples from intact sheep bone were not available. Therefore, we chose to compare the EST distribution in the PF7 and PF10 libraries to that of two resting human bone EST libraries. We reasoned that genes that were significantly more highly expressed in the sheep libraries could represent candidate fracture-healing related genes that might warrant closer investigation. A total of 39 genes with Benjamini-Hochberg-corrected *p*-values less than an arbitrary threshold of 0.05 were identified as putatively differentially expressed. Table 2 gives details on the genes and number of ESTs found (see Methods for abbreviations). Links to corresponding contigs in the GeneNest database are given in [Additional file 2].

It is striking that 24 of the 39 genes (62%) had previously been known to be involved with developmental processes in some way (references are given in the table). Eleven of the 24 genes were previously known to be involved in bone development or fracture healing. Given that fracture healing is thought to recapitulate bone development on a molecular level [9], these findings are suggestive that our EST analysis captured some essential aspects of fracture healing.

### Expression analysis of candidate fracture-healing related genes

We then reasoned that differential expression during the course of fracture healing could be another kind of indicator for genes with potential importance in fracture healing. Therefore, we performed RT-PCR expression analysis using samples from postfracture day 7, 10, 14, and 42. The RNA source for days 7 and 10 was identical to that used for the PF7 and PF10 EST libraries, and the source for day 14 was one of the RNA samples used to construct the pooled EST library. The day 42 RNA sample was not used for any of the EST libraries. Samples from normal sheep bone were not available.

PCR primers were designed for 30 of the genes in Table 2 and for an additional 48 candidate genes (Table 3), that were chosen for biological interest or because of tendencies toward overexpression that did not reach statistical significance.

Almost all genes showed a large drop in expression at the 42-day time point. Early stage callus tissue is a metabolically highly active tissue. In comparison, healthy bone is less metabolically active, which is not surprising given that cells make up only around 2% of the total mass of bone. It has previously been noted that the expression of many genes related to fracture healing drops to very low or undetectable levels at 6 weeks after fracture in a rat model [14]. In our experiments, 77 of the 78 genes (99%) tested by ANOVA over all four timepoints were found to be significantly differentially expressed, a proportion that dropped to 96% if only the first three time points were considered, and 31% for a comparison between only the first two time points. [Additional file 2] presents the normalized expression values determined in these experiments.

**Figure 6:**
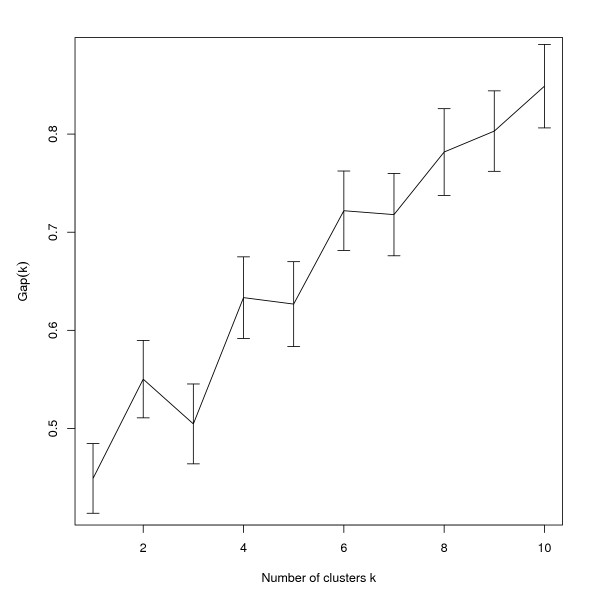
**Estimation of optimal cluster number by gap-stat analysis**. The gap statistic for the hierarchical clustering of the genes analyzed by RT-PCR. The within-cluster dispersion (an error measure) decreases monotonically as the number of clusters increases. If the data do tend to form a certain number of clusters, say *k*, then the decrease in the within-cluster dispersion will flatten markedly thereafter. The gap statistic calculates the value(s) of *k *at which this happens in order to estimate the optimal number of clusters [15]. In the RT-PCR data, two well separated clusters can be identified because of the local maximum at *k *= 2. However, the gap function rises again with a strong increase at *k *= 4 and *k *= 6, suggesting the existence of less well separated clusters within the main clusters.

**Figure 7:**
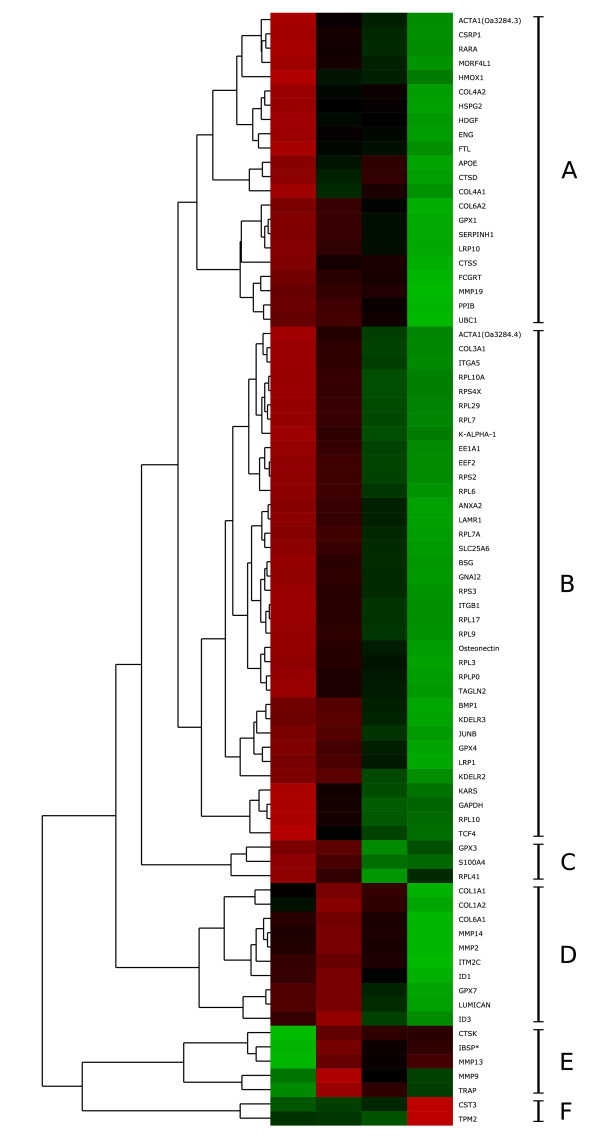
**Cluster analysis of RT-PCR expression values**. A dendogram showing expression profiles of the genes investigated by RT-PCR. For each of the 78 genes investigated by RT-PCR, expression values for 10d, 14d, and 42d were normalized to those at day 7 and clustered hierarchically by complete linkage (Values for IBSP were normalized to expression at day 10 because there was no detectable expression at day 7). The number of clusters was estimated by analysis with the gap-statistic (see Fig. 6). Six clusters A-F are shown.

We then performed hierarchical clustering to produce a graphic representation of the the expression patterns for the genes investigated in RT-PCR. We used the gap-stat method [15] to estimate the number of clusters in the data (Fig. 6). The estimated number of clusters is two. However, the gap function rises again with peaks at 4 and 6 clusters, suggesting that there are less well defined groups of four and of six clusters in the data. For the purposes of discussion and visualization, we chose to partition the genes into six clusters (Fig. 7). Clusters A, B, E and F demonstrated high frequencies of genes of specific functional classes (see below).

## Discussion

In the present work, we have performed large-scale EST sequencing and analysis in order to generate sufficient sequence information to study fracture healing at a genomic level in the sheep for the first time. The results of our study are of interest not only in the context of fracture healing and bone development, but will also provide useful information for all fields of research that use the sheep as a model organism. The ESTs generated in the present work represent an increase in the number of available ESTs by a factor of over 4.5, and provide at least partial sequences for over 5800 sheep genes never before sequenced. The sequences can be downloaded from the online supplemental site and are also available in the GeneNest Sheep database.

Genomic approaches to the sheep have great potential to accelerate progress in research areas using the sheep as a model organism. For instance, the first study using an ovine cDNA microarray prepared from unpublished EST libraries was very recently published. A number of genes differentially expressed in sheep resistant and susceptible to gastrointestinal nematode infection were identified [16].

Although we now have initial concepts of the molecular processes involved in fracture healing, many questions remain to be answered before a comprehensive picture of the genetic programs involved in fracture healing can emerge. An in-depth understanding of the genes involved in fracture healing and the regulatory mechanisms that control them will be prerequisite for developing optimal treatments for disturbances of fracture healing and will have relevance to other disorders such as osteoporosis. At present, we still do not have a comprehensive list of key genes and gene products involved in bone development and fracture healing. In this study, we have performed the first large-scale EST sequencing project using a model for fracture healing and have identified numerous differentially regulated genes not previously known to be involved in fracture healing. EST sequencing gives a nearly unbiased view of highly expressed sequences in a tissue (assuming that the process of producing the ESTs does not introduce a significant amount of bias), and therefore offers some advantages over candidate gene approaches and even over microarray-based approaches that may not cover the entire genome.

In this work, we have generated a profile of postfracture day 7 and day 10 genetic expression using EST libraries produced by automated plasmid purification. No normalization procedures were applied to select ESTs for sequencing. Therefore, we believe that the distribution of ESTs in these libraries gives a nearly unbiased view of genetic expression at these stages of fracture healing. In addition, we generated a third EST library from a pool of later stages of fracture healing and used RT-PCR to examine differential expression of 30 of the 39 genes found to be enriched in the PF7 and PF10 libraries and 48 other genes (Tables 2 and 3).

Previous genomic approaches to fracture healing have involved analysis of a rat femur fracture model based mainly on microarray expression analysis, but different experimental and statistical approaches were used [11,17,18]. These studies have collectively identified thousands of genes that are differentially expressed in fracture healing. The genes were grouped by the authors into a number of functional classes, which overlap to a large extent with the classes identified in the present investigation, including extracellular matrix proteins, ribosomal proteins, resorption/remodeling, transcriptional regulation and signal transduction. However, the specific genes belonging to the functional classes are often different among the three studies cited above as well as in comparison to our results. For instance, Hadjiargyrou and coworkers (2002) identified 20 differentially expressed ribosomal genes; in the present study, 16 differentially expressed ribosomal genes were identified. With the exception of ribosomal protein SA, there is no overlap between the two lists. This suggests that we are just beginning to identify to the full complement of genes involved in fracture healing.

### Genes differentially expressed in fracture healing

Many of the differentially expressed genes we identified were previously known to be involved in various biological processes involved in bone development or fracture healing (see Tables 2 and 3 for references), suggesting that our approach has captured some essential aspects of fracture healing. Additionally, we have demonstrated differential expression for many genes not previously known to be involved in fracture healing.

The differentially expressed genes can be divided into several different classes, as will be discussed below. Three of these classes, extracellular matrix proteins, resorption/remodeling/inflammation, and transcriptional regulation/signal transduction, have been well studied in the context of fracture healing, and our results have identified both previously known and novel differentially regulated genes with these functional roles.

In the following, we describe three other classes with numerous differentially regulated genes, angiogenesis, free-radical control, and ribosomal genes. These functional classes have received less attention to date, although they are known to be involved in the process of fracture healing. Fracture-related differential expression had not previously been shown for the great majority of genes in these classes (cf. Tables 2 and 3). These observations thus represent starting points for future studies on the role of processes such as angiogenesis and free-radical control in fracture healing.

#### Ribosomal genes

The single largest group of putatively upregulated genes in our analysis was made up of ribosomal proteins. There are over 75 ribosomal proteins [19], which are not necessarily expressed in concert [20]. We found 9 genes coding for ribosomal proteins to be significantly overexpressed in our analysis of EST distribution compared to normal human bone. All of these genes, as well as seven additional genes coding for ribosomal proteins (Table 3) showed differential expression in the RT-PCR analysis, with expression levels peaking at postfracture days 7 and 10 (Fig. 7). We additionally identified four translation factors as being overexpressed in our EST analysis (Table 2). RT-PCR for two of these genes confirmed differential expression. To the best of our knowledge, a specific role in fracture healing has not been identified for all but two of these genes to date, although some of them are known to be differentially expressed in certain developmental processes (Tables 2 and 3), and a number of other ribosomal genes are known to be differentially expressed in fracture healing [11].

Cluster B (Fig. 7) contained 36 genes. Interestingly, 13 of the 15 ribosomal genes analyzed by RT-PCR, and both of the translation factors analyzed by RT-PCR were in this cluster, whose expression levels peaked during the early phases of fracture healing at days 7 and 10 postfracture, suggesting that there is an especially high level of protein synthesis at these stages.

#### Extracellular matrix proteins

Several matrix proteins were found to be differentially regulated. The gene with the highest overall expression, Collagen I*α*1, is the main protein component of bone, and the fact that it is one of the most highly upregulated genes is not surprising. Two other collagens and osteonectin were also found to be upregulated by EST analysis (Table 2). We investigated these genes and four other collagen genes and two proteoglycans by RT-PCR and showed differential expression for all genes. In general, the expression of these matrix genes was highest at postfracture day 7 or 10 but was still relatively high at day 14. IBSP is an osteoblast marker that is a major noncollagenous structural protein of the bone matrix. IBSP showed an expression pattern different from that of the other matrix genes, with significant expression beginning at day 10, peaking at day 14, and continuing until day 42.

The lysyl-tRNA synthetase KARS has no known involvement in fracture healing. Given the important role of lysine crosslinks in the biosynthesis of collagen, it is interesting that lysyl-tRNA synthetase was the only tRNA synthetase gene that was overexpressed in our data.

SERPINH1 was also found to be significantly overexpressed in the PF7 and PF10 ESTs. It is a molecular chaperone involved in the maturation of collagen molecules [21]. This is presumably important for bone development because SERPINH1 is localized to regions of type I collagen production in developing murine femurs and tibiae [22,23].

#### Angiogenesis-related genes

It has long been known that vascular invasion is necessary for bone differentiation [24], and angiogenesis is a key process for fracture healing [25]. A number of genes related to angiogenesis have previously been shown to be upregulated during bone repair, such as vascular endothelial growth factor [26].

COL3A1 and COL4A1 were shown to be overexpressed in the PF7 and PF10 ESTs (Table 2). Both of these collagens have known roles in angiogenesis [27,28].

Six of the eight genes in our dataset with prominent roles in angiogenesis (CSRP1, COL4A1, COL4A2, Perlecan, HDGF, Endoglin) were in cluster A. The smooth muscle marker CSRP1 [29] and the TGF*β*-family auxiliary receptor endoglin are required for angiogenesis and heart development [30,31]. HDGF is a highly expressed vascular endothelial cell protein [32].

The genes of this cluster show especially strong expression on day 7 postfracture with lesser amounts of expression on days 10 and 14, which is not surprising given that new blood vessel formation is a prerequisite for the formation of new bone from callus tissue. The other two genes, COL3A1 and TAGLN2, were in cluster B, which like cluster A is characterized by high early expression (PF7 and PF10), but shows a stronger drop in expression at the 14 day timepoint.

#### Resorption, remodeling and inflammation

Several genes that probably play a role in the inflammatory response characteristic of early stages of fracture healing (FCGRT, Basigin, HLA-A, and CD74) were found to be overexpressed in the PF7 and PF10 ESTs (Table 2).

Resorption of cartilage and bone mediated by osteoclasts is an important part of the remodeling processes involved in new bone formation in fracture healing. The main enzymes involved are thought to be the cathepsins and the matrix metalloproteinases (MMPs), which act in a concerted fashion. Although not all details are clear, our current understanding of this process suggests that osteoclasts create an acidic environment in their resorption lacunae, which results in dissolution of the mineral, and then secrete cysteine proteinases that are active at a low pH, to initiate proteolysis of the proteinaceous bone matrix. Finally, MMPs exert their activity, once the pH has increased sufficiently [33]. Our data has demonstrated differential expression for several enzymes from both classes.

Interestingly, four of the five genes in RT-PCR cluster E encode enzymes with roles in osteogenesis (ACP5, CTSK, MMP9, and MMP13). The first three genes are expressed by osteoclasts. The expression of genes in this cluster was minimal at day 7 but peaked at day 10 postfracture, suggesting that these enzymes might be particularly important for the processes involved in the bone formation that becomes visible by day 10.

We showed differential expression for MMP2, MMP9, MMP13, MMP14, and MMP19. All of these MMPs had previously known roles in bone development except MMP19, providing further evidence for the similarity of bone development and fracture healing. MMP2, MMP9, and MMP14 are differentially regulated in scarless fetal wound healing [34] and during osteogenesis [35], which is interesting because bone is one of the few adult tissues that can heal without scarring. MMP19 previously had no known role in skeletal development or fracture healing, but it is highly expressed in dermal wounds, suggesting a role in wound repair [36]. The highest expression of MMP19 was observed in the day 7 and 10 stages.

Cluster F contained only two genes, cystatin C and TPM2. These genes were the only ones with an expression profile that peaked at the last time point (42 days postfracture). Cystatin C (CST3) can inhibit bone resorption and osteoclast formation [37]. Osteoclast activity begins early and increases over the course of fracture healing [38], and one may speculate that CST3 may be important for regulation of osteoclasts at this time point.

#### Control of free radicals

Vascular invasion of ossified cartilage during enchondral ossification is associated with breakdown of tissue by the release of lytic enzymes by invading cells such as macrophages and endothelial cells. This process is likely to involve the production of reactive oxygen species (ROS) [39]. At present, little is known about the mechanisms by which unwanted tissue damage by ROS is controlled during fracture healing. The glutathione peroxidases (GPX) are enzymes that catalyze the reduction of hydrogen peroxide, organic hydroperoxide, and lipid peroxides by reduced glutathione. They are thus involved in the protection of cells against oxidative damage. Although none of the four GPX genes we tested in RT-PCR were individually statistically significantly overexpressed in the EST analysis, there were no ESTs for any of these GPX genes in normal bone and a total of 85 ESTs for the four GPX genes in the PF7 and PF10 EST libraries. We showed significant differential expression patterns for each of these genes by RT-PCR, although they did not show a uniform expression pattern (Fig. 7).

HMOX1 was also found to be significantly overexpressed in the PF7 and PF10 EST libraries, as has been previously described for postfracture day 3 in a rat femur fracture model [40]. HMOX1 is an inducible heme oxygenase that could conceivably be involved in heme catabolism during resorption of the initial fracture hematoma, although other roles in development have been postulated for this gene [41].

#### Transcription regulation and signal transduction

Two membrane-bound proteins with roles in signal transduction were found to be significantly overexpressed in the PF7 and PF10 libraries. ITM2C is highly expressed during chondro-osteogenic differentiation [42], but its specific function and its role in fracture healing remain unknown. The G protein GNAI2 was also found to be overexpressed, but no clues are available as to its particular role in fracture healing.

Signal transduction and modification of transcriptional programs by transcription factors represent important phenomena in development, and presumably are essential for fracture healing. Nevertheless, the EST counts for the transcription factors examined in this study were too low to reach statistical significance, even though in many cases ESTs for transcription factors were found only in the PF7 and PF10 libraries but not in the human normal bone libraries. For instance, we identified only one EST each for the transcription factor runx-2 and osterix, which are both key players in skeletogenesis [43,44].

We performed RT-PCR analysis on a number of transcription factors with relatively large numbers of ESTs in our libraries (Table 3). We were able to demonstrate differential expression with the highest expression at days 7 and 10, for JunB, which among other roles is a positive regulator controlling primarily osteoblast as well as osteoclast activity [45]. MORF4L1, which may play a role in mechanotransduction [46], and TCF4, which may mediate Wnt signaling during limb development [47] also showed highest expression levels in the first two time points. Additionally, we showed differential expression for NSEP1, PPIB, and RARA, for which to the best of our knowledge no previous information about differential expression during bone development or fracture healing was available.

## Conclusion

In this work we present approximately 47,000 ESTs from the domestic sheep. This represents an important resource to researchers interested in the sheep as a model organism and greatly expands the number of gene sequences available for *Ovis aries *.The sequences are freely available in GeneNest and the public sequence databases. We have used the sequences we generated as a tool to investigate the early stages of fracture healing and have identified numerous genes that are differentially regulated in early callus tissues, many of which were not previously known to have any specific relation to fracture healing or bone development. These genes will be valuable leads for further research into the molecular mechanisms of fracture healing.

## Methods

### Fracture model

All animal experiments were carried out according to the policies and principles established by the Animal Welfare Act, the NIH Guide for Care and Use of Laboratory Animals and the national animal welfare guidelines. The study was approved by the local legal representative (Landesamt für Arbeitsschutz, Gesundheitsschutz und technische Sicherheit, Berlin: G 0224/01 and G 0172/04).

The experimental system has been described in detail elsewhere [48,49,38]. Briefly, twelve two-year old female Merino mix sheep with a mean weight of 66 ± 12 kg underwent a standardized midshaft osteotomy of the right tibia under general anesthesia. The osteotomy was distracted to a gap of 3 mm and stabilized with a monolateral external fixator. The fixator consisted of 6 Schanz screws (5 mm, 3 inserted proximally, 3 inserted distally of the osteotomy) and 2 steel tubes (10 mm) and was mounted medially to stabilize the defect.

The osteotomy wound was sutured and the leg, together with the external fixator, was covered with a tube bandage. The animals received an analgetic (Finadyne, Essex, Germany) for 3 days postoperatively. Daily pin care involved cleaning of the insertions of the Schanz screws with ethacridin lactate (Rivanol, 5%, Chinosol, Germany).

After removal of the fixator under general anesthesia the callus tissue was removed and sheep were sacrificed. The tissue was frozen in liquid nitrogen and stored at -80°C until further use.

### Isolation of RNA and library construction

For generation of ESTs, three separate libraries were constructed. The first library (PF7) was isolated from four animals 7 days post-fracture, the second library (PF10) was isolated from three animals 10 days post-fracture, and the third library was isolated from a pool of one animal each at 2 weeks, 3 weeks, and 4.5 weeks post-fracture.

PF7 consisted of 3.6 × 10^5 ^clones with an average insert length of 1.6 kb. PF10 consisted of 3.4 × 10^5 ^clones with an average insert length of 1.4 kb. The pooled library consisted of 4.5 × 10^6 ^clones with an average insert size of 1.4 kb. Average insert lengths were determined by screening at least 300 inserts per library.

For isolation of RNA the tissue was ground in a liquid-nitrogen-cooled stainless steel mortar. RNA was isolated with peqGOLD TriFast (peqLab) and subsequently purified with RNeasy Mini columns (Qiagen) followed by poly(A)-RNA isolation using the polyATtract mRNA Isolation System III (Promega).

For construction of cDNA libraries the CloneMiner cDNA library construction Kit (Invitrogen) was used according to the manufacturer's protocol with the following modifications. All precipitations were carried out using PelletPaint Co-Precipitant (Novagen) instead of glycogen. The first strand reaction was carried out in five separate reactions with 1 *μ*g poly(A)-RNA each. Four reactions were precipitated and pooled with the fifth for a single second strand reaction. Glass fiber filters for radioactive determination of the cDNA yield were processed separately. Clones for further analysis were arrayed into 384-well-microtiter plates by robotic colony picking.

### Sequencing and analysis of ESTs

Template DNA for sequencing was prepared by PCR amplification of cDNA inserts with M13 primers or by automated plasmid purification. PCR product quality control was done by 384 well agarose gels, positive products where rearrayed and diluted to appropriate concentrations. For plasmid preparation, cultures were grown in 384 deepwell plates containing 200 *μ*l of 2YT Media. Incubation took 17–18 h at 1100 rpm.

Plasmids were isolated and purified automatically by a modified alkaline lysis procedure. Quality control of plasmids was done by randomly testing eight samples of each plate on agarose gels. Sequences were determined using M13 -40 primer, ABI Big Dye Terminator 3 chemistry and ABI 3730XL capillary sequencer systems (ABI, Weiterstadt, Germany). All raw sequences were processed by PHRED [50,51] and screened for vector or *Escherichia coli *contamination.

Before clustering, sheep EST sequences were subjected to an extensive quality control procedure. In a first step sequences were clipped at both ends as long as PHRED quality values stayed below 20 in a window of size 15 bp. Secondly, for all sequences (ESTs + mRNAs) putative vector and repeat sequences were masked. Clustering and assembly of the clipped sequences was performed using the GeneNest software tools [12,13].

### An *Ovis aries *EST database

GeneNest is designed to generate and visualize gene indices based on ESTs and mRNA sequences [12,13]. We have used the GeneNest software to create a database with 47209 sheep ESTs generated in the current project, as well as all previously available *Ovis aries *sequences. The database is available at .

### Accession numbers

All ESTs generated in this project were submitted to NCBI's GenBank and are available with the accession numbers [GenBank:DY475559–DY505707] and [GenBank:DY505714–DY522957].

### Identification and annotation of genes

In order to identify and annotate novel and previously known sheep genes, similarities to known proteins were identified with a blastx [52] search against the RefSeq protein database [53] of 28 September, 2005 using default settings for blastx. Contigs with a best e-value of 10^-25 ^or lower were assigned a putative identity.

### Gene Ontology

For the purpose of Gene Ontology [54] annotation, the contigs were mapped to mouse mRNAs using blastn with default settings. Putative identities were assigned using an e-value threshold of 10^-25^. Mouse-Genome Informatics (MGI) annotations [55] were assigned to the sheep homologs of annotated mouse genes, and GO analysis was performed using the Ontologizer [56].

### Differential expression of early fracture genes

In order to gain insight into the genes that are upregulated in the early stages of fracture healing in the sheep, we compared the distribution of ESTs in the PF7 and PF10 sheep libraries against the distribution of ESTs in the UniGene [6] human bone libraries ID.655 [57] and ID. 1124, which have a total of 6573 ESTs. Mapping of sheep contigs to human UniGene clusters was performed using blastn with an e-value cutoff of 10^-25 ^as described above. We then compared the EST counts for the PF7/PF10 libraries against those in the ID.655/ID.1124 libraries using a two-tailed Fisher-Exact test. The result of the Fisher-exact test is a *p*-value that reflects the probability of observing the given differences in EST counts in the respective libraries assuming no differential expression [58]. The expression ratio was calculated based on the total counts of 32032 ESTs in libraries PF7 and PF10 and 6573 ESTs in UniGene libraries ID.655/ID.1124. A similar test was applied to compare EST counts in PF7 against those in PF10. If the *p*-value of this association test is below a certain threshold, the gene is considered as putatively differentially expressed. Correction for multiple testing was performed using a false discovery rate (FDR)-control method [59]. The FDR represents the expected proportion of errors among the rejected null hypotheses and is thus a different kind of procedure from multiple testing correction procedures such as a Bonferroni correction that control the family-wise error rate, or the probability that one or more rejected null hypotheses are falsely rejected. The Benjamini-Hochberg-adjusted *p*-values are shown in the Tables for the EST comparisons.

### RT-PCR

RNA was isolated for quantitative reverse-transcriptase polymerase chain reaction (RT-PCR) from 7-day postfracture (PF7, n = 4 animals), 10-day postfracture (PF10, n = 3), 14 day (PF14, n = 1) and 42-day (PF42, n = 1) tissue. PF7 and PF10 were identical to the RNA sources used for EST library construction, and PF14 was used for the pooled library. For semi-quantitative RT-PCR measurements, single-stranded cDNA was prepared from the different healing stages using random hexamer primers with the Taqman Reverse Transcription Reagents Kit (Applied Biosystems, Forster City, USA). Gene-specific primers were designed using the Primer Express software (Applied Biosystems) and quantitative RT-PCR was performed with SYBR green PCR Master Mix (Applied Biosystems) on an ABI Prism 7900 HT (Applied Biosystems). The mRNA levels were quantified in triplicate by the standard curve method normalized to a exogenous green fluorescent protein (GFP) transcript of which 1 ng was spiked into the sample per 1 *μ*g of total RNA. See [Additional file 2] for primer sequences and RT-PCR profiles.

Three measurements were made for each gene at each of the four time points. Statistical analysis of the RT-PCR results was performed by a two-sided Student's *t *test to compare expression between PF7 and PF10, and one-way ANOVA was performed to identify differential expression among the groups PF7, PF10, PF14 or among all four groups. A Bonferroni correction for multiple testing was performed.

### Clustering and visualization of RT-PCR results

Complete-linkage hierarchical clustering was used to visualize the results of RT-PCR on the 78 putative fracture-related genes. For each gene, a vector of four values representing the four time points was normalized to have zero mean and unit variance. In order to estimate the optimal number of clusters, the gap statistic method [15] was used.

## Abbreviations

ACP5 = Acid phosphatase 5, tartrate resistant; ACTA1 = Actin, *α*1; ANXA2 = Annexin A2; APOE = Apolipoprotein E; BMP1 = Bone morphogenetic protein 1; CD74 = CD74 antigen; COL = Collagen (COL1A1, COL1A2, COL3A1, COL4A1, COL4A2, COL6A1, COL6A2); COX1 = Cytochrome c oxidase subunit I; CSRP1 = Cysteine and Glycine-Rich Protein 1; CST3 = Cystatin C; CTS = Cathepsin (CTSD, CTSK, CTSS); EEF = Eukaryotic translation elongation factor (EEF1A1, EEF2) EIF = Eukaryotic translation initiation factor (EIF4A1, EIF5A); ENO1 = Enolase 1; FCGRT = Fc fragment of IgG, receptor, transporter, *α*; FTH1 = Ferritin, heavy polypeptide 1; FTL = Ferritin, light polypeptide; GAPDH = glyceraldehyde-3-phosphate dehydrogenase; GNAI2 = G protein, *α *inhibiting activity polypeptide 2; GPX = Glutathione peroxidase (GPX1, GPX3, GXP4, GPX7); HDGF = Hepatoma-derived growth factor; HLA-A = Major histocompatibility complex, class I, A; HMOX1 = Heme oxygenase (decycling) 1); IBSP = Integrin-binding sialoprotein; ID = DNA-binding protein inhibitor (ID1, ID3); ITGA5 = Integrin, alpha 5; ITGB1 = Integrin, beta 1; ITM2C = Integral membrane protein 2C; JUNB = Jun B proto-oncogene; K-ALPHA-1 = *α *Tubulin, ubiquitous; KARS = Lysyl-tRNA synthetase; KDELR = KDEL (Lys-Asp-Glu-Leu) endoplasmic reticulum protein retention receptor (KDELR2, KDELR3); LAMR1 = Ribosomal protein SA; LRP = LDL receptor-related protein (LRP1, LRP10); MORF4L1 = Mortality factor 4 like 1; MMP = Matrix metalloproteinase (MMP2, MMP9, MMP13, MMP14, MMP19); NSEP1 = Nuclease-sensitive element-binding protein 1; PGK1 = phosphoglycerate kinase 1; PPIB = Peptidylprolyl isomerase B (cyclophilin B); RARA = Retinoic acid receptor, alpha; RPL = Ribosomal protein L (RPL3, RPL6, RPL7, RPL7A, RPL9, RPL10, RPLlOa, RPL17, RPL29, RPL41, RPLPO); RPS = Ribosomal protein S (RPS2, RPS3, RPS4X, RPS5); S100A4 = S100 calcium binding protein A4; SERPINH1 = Serpin peptidase inhibitor, clade H, member 1; SLC25A6 = Solute carrier family 25 member 6; TAGLN2 = Transgelin 2; TCF4 = Transcription factor 4; TPM2 = *β *Tropomyosin 2; UBC = Ubiquitin C

## Authors' contributions

JH performed and contributed to the design of the biological experiments with the help of ACS and VS. HK and AJP performed the EST sequencing under the guidance of RR. SAH analyzed and clustered the ESTs using GeneNest. SB and PNR were responsible for the bioinformatic analysis. JL and HS performed experiments on the sheep model under the guidance of GND. SM conceived and designed the study. PNR wrote the manuscript with the help of SB and JH. All authors read and approved the final manuscript.

## Supplementary Material

Additional file 1 A gzip-compressed FASTA file containing sequences of the 19087 contigs described in this study. Click here for file

Additional file 2 A complete list of sheep genes upregulated in early fracture healing as determined by EST analysis with links to human homologs in UniGene and to the GeneNest database. Additionally, RT-PCR primer sequences and detailed results are shown. Click here for file

## Figures and Tables

**Table 1:** EST counts of the libraries.

Library	ESTs	Number of contigs
PF7	14884	5859
PF10	17148	6217
Pooled	15177	5421

**Table 2:** Significantly overexpressed genes in early fracture tissue. The columns Gene and UniGene show the symbol and UniGene cluster id for the human ortholog of the sheep gene in question. See Methods for abbreviations. The genes are ordered according to Benjamini-Hochberg (BH)-corrected *p*-value. EST Ratio shows the EST counts from PF7/PF10 vs. the counts in the resting human bone libraries; the adjusted count ratio is given in parentheses followed by the BH-corrected *p*-value. The adjusted count ratio was calculated by the formula *ad/bc*, where *a *was the count of a given gene in the PF7/PF10 libraries, *b *the total count of ESTs in PF7/PF10, *c *the count of the gene in the human EST libraries, and *d *the total count of ESTs in the human libraries. That is, the EST counts of individual genes were corrected for the total number of ESTs in the EST libraries to provide information about the relative abundance of ESTs corresponding to a given gene. RT-PCR summarizes the statistical analysis of the RT-PCR results for the comparison between PF7 and PF10 (first entry), among PF7, PF10, and PF14 (second entry), and among PF7, PF10, PF14, and PF42 (third entry). '-' indicates non-significant, '*' indicates an adjusted *p*-value of 0.05 > *p *> 10^-4^, and "**" indicates a *p*-value less than 10^-4^. "n.d.", not done. A complete list of absolute fold change values for all genes is available in the online supplement. Bone/Development indicates whether the gene in question has previously been shown to be involved in bone development or fracture healing (first entry) or development (second entry). See Methods for the abbreviations. §No values were available for the 14d time point for FTH1.

**Gene**	**Unigene**	**EST Ratio**	**RT-PCR**	**Bone/Development**
EEF1A1	Hs.522463	664:32 (4.3; *p *= 5.7 × 10^-21^)	-/*/**	+/+ [60]
COX1	Hs.550345	265:0 (-;*p *= 4.0 × 10^-19^)	n.d.	-/+ [61]
RPLPO	Hs.448226	278:1 (57; *p *= 1.3 × 10^-18^)	-/**/**	-/-
COL1A1	Hs. 172928	1987:249(1.6; *p *= 5.3 × 10^-13^)	*/**/**	+/+ [62]
Osteonectin	Hs.111779	588:39(3.1; *p *= 2.9 × 10^-12^)	-/*/**	+/+ [63]
RPS2	Hs.506997	268:7 (7.9; *p *= 8.6 × 10^-12^)	*/**/**	-/-
FTH1^§^	Hs.558804	332:14 (4.9; *p *= 3.7 × 10^-11^)	-/-/**	+/+ [60]
RPL3	Hs.119598	179:1 (37; *p *= 4.4 × 10^-11^)	-/*/**	-/+ [64]
LAMR1	Hs.558354	221:4 (11; *p *= 4.9 × 10^-11^)	-/**/**	+/+ [11]
UBC	Hs.520348	190:3 (13; *p *= 8.5 × 10^-10^	*/**/**	-/-
ITM2C	Hs.111577	114:0 (-;*p *= 1.4 × 10^-7^)	-/**/**	+/+ [42]
COL3A1	Hs.443625	444:42 (2.2; *p *= 1.8 × 10^-5^)	-/**/**	-/+ [65]
EEF2	Hs.515070	85:0(-;*p *= 2.9 × 10^-5^)	-/**/**	-/+ [66]
COL4A1	Hs.17441	105:2 (11; *p *= 9.5 × 10^-5^)	-/*/*	-/+ [67]
RPL7A	Hs.558380	78:0(-;*p *= 9.6 × 10^-5^)	-/*/**	-/+ [68]
K- ALPHA- 1	Hs.334017	89:1 (18; *p *= 0.00016)	*/**/**	-/-
ACTA1	Hs.1288	72:0 (-;*p *= 0.00021)	*/**/**	-/-
SLC25A6	Hs.350927	71:0(-;*p *= 0.00032)	-/**/**	-/+ [69]
EIF4A1	Hs. 129673	82:1(17; *p *= 0.00047)	n.d.	-/-
HMOX1	Hs.517581	66:0 (-;*p *= 0.00066)	*/**/**	-/+ [41]
FTL	Hs.433670	63:0 (-; *p *= 0.00096)	-/*/**	-/+ [70]
RPL7	Hs.421257	63:0 (-; *p *= 0.00096)	-/**/**	-/-
ACP5	Hs.1211	58:0 (-;*p *= 0.0019)	*/**/**	+/+ [71]
SERPINH1	Hs.241579	154:10 (3.2; *p *= 0.0038)	*/**/**	+/+ [22]
GNAI2	Hs.77269	52:0 (-; *p *= 0.0072)	-/**/*	-/+ [72]
RPS5	Hs.378103	62:1 (18; *p *= 0.0080)	n.d.	-/-
EIF5A	Hs.534314	73:2 (7.5; *p *= 0.0083)	n.d.	-/-
RPS3	Hs.546286	50:0 (-;*p *= 0.0089)	-/**/**	-/-
MMP14	Hs.2399	47:0 (-;*p *= 0.013)	**/**/**	+/+ [73]
APOE	Hs.515465	47:0 (-; *p *= 0.013)	-/-/*	-/-
ENO1	Hs.517145	146:11 (2.7; *p *= 0.017)	n.d.	+/+ [60]
HLA-A	Hs.181244	88:4 (4.5; *p *= 0.018)	n.d.	-/-
KARS	Hs.3100	44:0 (-;*p *= 0.019)	-/*/*	-/-
Basigin	Hs.501293	45:0 (-;*p *= 0.019)	-/*/**	-/+ [74]
CD74	Hs.436568	56:1 (11; *p *= 0.024)	n.d.	-/+ [75]
RPL10	Hs.401929	43:0 (-; *p *= 0.025)	-/**/**	+/+ [76]
PGK1	Hs.78771	54:1 (11; *p *= 0.026)	n.d.	-/-
FCGRT	Hs.111903	40:0 (-;*p *= 0.031)	-/*/**	-/-
NSEP1	Hs.512451	50:1 (10; *p *= 0.036)	n.d.	-/+ [77]

**Table 3:** Supplemental genes investigated by RT-PCR for differential xpression. See Methods for abbreviations and Table 2 for explanations.

**Gene**	**UniGene**	**EST Ratio**	**RT-PCR**	**Bone/Development**
COL1A2	Hs.489142	522:210 (0.51; *p *= 1.2 × 10^-12^)	*/**/**	+/+ [60]
ITGB1	Hs.429052	8:21 (0.078; *p= *1.5 × 10^-8^)	-/**/**	+/+ [78]
COL6A2	Hs.420269	111:56 (0.41; *p *= 2.8 × 10^-5^)	-/**/**	+/+ [79]
TAGLN2	Hs.517168	110:8 (2.8; *p *= 0.054)	-/**/**	-/-
CTSS	Hs.181301	34:0 (-; *p *= 0.069)	-/**/**	-/+ [80]
MORF4L1	Hs.374503	34:0 (-; *p *= 0.069)	-/*/**	-/-
GPX3	Hs.386793	36:0 (-; *p *= 0.073)	-/**/**	+/+ [17]
Endoglin	Hs.76753	32:0 (-; *p *= 0.087)	-/*/**	-/+ [30]
RPL9	Hs.412370	32:0 (-; *p *= 0.087)	-/**/**	-/+ [81]
CSRP1	Hs.108080	95:7 (2.8; *p *= 0.089)	-/**/**	-/+ [29]
RPS4X	Hs.446628	31:0 (-; *p *= 0.11)	-/*/**	-/-
RPL10a	Hs.546269	30:0 (-; *p *= 0.11)	-/**/**	-/-
RPL6	Hs.546283	30:0 (-; *p *= 0.11)	-/**/**	-/-
PPIB	Hs.434937	31:0 (-;*p *= 0.11)	-/**/**	+/+ [60]
ANXA2	Hs.511605	158:17 (1.9; *p *= 0.14)	*/**/**	+/+ [82]
JUNB	Hs.25292	27:0 (-;*p *= 0.15)	-/**/**	+/+ [83]
RPL17	Hs.374588	27:0 (-;*p *= 0.15)	-/**/**	-/-
TPM2	Hs.300772	28:0 (-;*p *= 0.16)	-/**/**	-/-
Lumican	Hs.406475	114:12 (1.9; *p *= 0.21)	*/**/**	+/+ [84]
MMP2	Hs.513617	53:20 (0.54; *p *= 0.21)	*/**/**	+/+ [85]
Perlecan	Hs.555874	45:2 (4.6; *p *= 0.21)	*/**/**	+/+ [86]
RPL41	Hs.381172	0:2 (-;*p *= 0.21)	*/**/**	-/-
GPX1	Hs.76686	20:0 (-;*p *= 0.26)	*/**/**	-/-
MMP13	Hs.2936	7:5 (0.29; *p *= 0.27)	**/**/**	+/+ [87]
GAPDH	Hs.544577	137:17 (1.7; *p *= 0.36)	-/*/**	-/-
RARA	Hs.535499	9:0 (-;*p *= 0.37)	-/*/*	+/+ [88]
ITGA5	Hs.505654	16:0 (-;*p *= 0.51)	*/**/**	+/+ [89]
BMP1	Hs.1274	16:0 (-;*p *= 0.51)	-/**/**	+/+ [90]
GPX7	Hs.43728	16:0 (-;*p *= 0.51)	-/**/**	-/-
LRP1	Hs. 162757	16:7 (0.47; *p *= 0.53)	-/**/**	-/-
COL4A2	Hs.508716	73:8 (1.9; *p *= 0.56)	*/**/**	-/-
GPX4	Hs.433951	13:0 (-;*p *= 0.59)	-/**/**	-/-
IBSP	Hs.518726	8:4 (0.41; *p *= 0.59)	**/**/**	+/+ [11]
RPL29	Hs.425125	0:1 (-;p = 0.59)	*/**/**	-/-
S100A4	Hs.81256	13:0 (-;*p *= 0.59)	-/**/**	+/+ [91]
ID1	Hs.504609	7:0 (-;*p *= 0.61)	-/*/*	+/+ [92]
KDELR2	Hs.520210	10:0 (-;*p *= 0.76)	-/**/**	-/-
MMP9	Hs.297413	11:0 (-;*p *= 0.76)	**/*/**	+/+ [35]
LRP10	Hs.525232	9:4(0.46; *p *= 0.83)	-/*/*	-/-
HDGF	Hs.506748	14:5 (0.57; *p *= 1.0)	-/*/*	-/+ [32]
COL6A1	Hs.474053	159:29 (1.1; *p *= 1.0)	-/-/*	+/+ [79]
CTSD	Hs.546248	87:13 (1.4; *p *= 1.0)	**/**/**	-/-
CTSK	Hs.523594	27:4 (1.4; *p *= 1.0)	*/**/**	+/+ [93]
ID3	Hs.76884	13:2 (1.3; *p *= 1.0)	-/*/*	+/+ [92]
KDELR3	Hs.554798	l:0 (-; *p *= 1.0)	-/**/**	-/-
MMP19	Hs. 154057	16:3 (1.1; *p *= 1.0)	-/**/**	-/-
TCF4	Hs.200285	5:0 (-;p = 1.0)	*/*/**	+/+ [47]
CST3	Hs.304682	12:2 (1.2; *p *= 1.0)	-/**/**	+/+ [37]
