# Supplementary material for: Gene identification and analysis of transcripts differentially regulated in fracture healing by EST sequencing in the domestic sheep
Source: BMC Genomics. 2006 Jul 5;7:172. doi: 10.1186/1471-2164-7-172 (PMC1578570; doi:10.1186/1471-2164-7-172)
Supplement: Additional file 2 — A complete list of sheep genes upregulated in early fracture healing as determined by EST analysis with links to human homologs in UniGene and to the GeneNest database. Additionally, RT-PCR primer sequences and detailed results are shown. [file 1471-2164-7-172-S2.HTML]

Gene identification and analysis of transcripts differentially regulated in
fracture healing by EST sequencing in the domestic sheep:
Online supplement

## Gene identification and analysis of transcripts differentially regulated in fracture healing by EST sequencing in the domestic sheep: Online supplement

### Jochen Hecht

Max Planck Institute for Molecular Genetics, Berlin, Germany

### Heiner Kuhl

Max Planck Institute for Molecular Genetics, Berlin, Germany

### Stefan A. Haas

Max Planck Institute for Molecular Genetics, Berlin, Germany

### Sebastian Bauer

Institute for Medical Genetics, Charite Universit�tsmedizin, Berlin, Germany

### Albert J. Poustka

Max Planck Institute for Molecular Genetics, Berlin, Germany

### Jasmin Lienau

Center for Musculoskeletal Surgery, Charite Universit�tsmedizin, Berlin, Germany

### Hanna Schell

Center for Musculoskeletal Surgery, Charite Universit�tsmedizin, Berlin, Germany

### Asita C. Stiege

Max Planck Institute for Molecular Genetics, Berlin, Germany

### Volkhard Seitz

Max Planck Institute for Molecular Genetics, Berlin, Germany

### Richard Reinhardt

Max Planck Institute for Molecular Genetics, Berlin, Germany

### Georg Duda

Center for Musculoskeletal Surgery, Charite Universit�tsmedizin, Berlin, Germany

### Stefan Mundlos

Institute for Medical Genetics, Charite Universit�tsmedizin, Berlin, Germany

### Peter N Robinson

Institute for Medical Genetics, Charite Universit�tsmedizin, Berlin, Germany

`<peter.robinson@charite.de>`

---

**Table of Contents**

Sheep Genes Upregulated in Early Fracture Healing as Determined by EST Analysis

RT-PCR Primer

RT-PCR Results

Partial or
complete sequence information for numerous previously uncharacterized
sheep genes were identified in the course of this project. All
sequences have been deposited in public sequence repositories and are
also available at the GeneNest database. A putative identity could be assigned for about 5,800 of these genes
on the basis of e-values of 10-25 or less by BLAST analysis to a set of protein RefSeqs. A separate
FASTA file containing these sequences is available here.

## Sheep Genes Upregulated in Early Fracture Healing as Determined by EST Analysis

The
following tables offer a listing of the genes that were found to be
overexpressed by Fisher's Exact Test of EST data (see manuscript for
details). The table shows the presumptive identity of the sheep cluster
and lists the corresponding contigs upon which the clusters are based.
The sequences of the contigs can be found at the GeneNest database.

**Table 1.**

|  |  |  |  |
| --- | --- | --- | --- |
| Gene | Symbol | *H. sapiens* homolog | *Ovis aries* contig |
| Cytochrome c oxidase subunit I; | COX1 | Hs.550345 | Oa3043.1 |
| Eukaryotic translation elongation factor 1 alpha 1; | EEF1A1 | Hs.522463 | Oa3247.1 |
| Ribosomal protein L P0 | RPLP0 | Hs.448226 | Oa3428.1 & Oa3428.2 |
| Osteonectin | SPARC | Hs.111779 | Oa437.2 & Oa437.6 & Oa437.8 |
| Collagen I, alpha 1 | COL1A1 | Hs.172928 | Oa517.4 & Oa2002.1 & Oa2002.6 & Oa2002.7 & Oa2002.8 & Oa2002.9 & Oa2002.10 & Oa2002.11 & Oa2002.12 & Oa2002.13 & Oa2002.14 & Oa2002.15 & Oa2002.16 & Oa2002.17 & Oa2002.18 & Oa2002.19 & Oa2002.20 & Oa2002.22 & Oa2002.24 & Oa2002.29 |
| Ribosomal protein S2 | RPS2 | Hs.506997 | Oa3320.1 |
| Ferritin, heavy polypeptide 1 | FTH1 | Hs.558804 | Oa2373.1 & Oa2373.3 |
| Integral membrane protein 2C | ITM2C | Hs.111577 | Oa5278.1 & Oa5278.2 & Oa5278.5 |
| Ribosomal protein L3 | RPL3 | Hs.119598 | Oa691.1 & Oa13692.1 |
| Ribosomal protein SA | LAMR1 | Hs.558354 | Oa829.1 |
| Ubiquitin C | UBC | Hs.520348 | Oa4596.1 & Oa4596.2 |
| Eukaryotic translation elongation factor 2 | EEF2 | Hs.515070 | Oa3405.1 & Oa3405.2 |
| Ribosomal protein L7a | RPL7A | Hs.558380 | Oa2982.1 |
| Actin, alpha 1, skeletal muscle | ACTA1 | Hs.1288 | Oa3284.3 & Oa3284.4 |
| Solute carrier family 25 (mitochondrial carrier; adenine nucleotide translocator), member 6 | SLC25A6 | Hs.350927 | Oa5193.1 |
| Heme oxygenase 1 | HMOX1 | Hs.517581 | Oa1094.1 |
| Ferritin, light polypeptide | FTL | Hs.433670 | Oa753.1 |
| Ribosomal Protein L7 | RPL7 | Hs.421257 | Oa1986.1 |
| Acid phosphatase 5, tartrate resistant | ACP5 (a.k.a. TRAP) | Hs.1211 | Oa2093.1 |
| Guanine nucleotide binding protein (G protein), alpha inhibiting activity polypeptide 2 | GNAI2 | Hs.77269 | Oa3850.1 & Oa3850.2 & Oa3850.3 |
| Ribosomal protein S3 | RPS3 | Hs.546286 | Oa943.1 & Oa1224.1 & Oa4417.1 |
| Matrix metallopeptidase 14 (membrane-inserted) | MMP14 | Hs.2399 | Oa4537.1 & Oa4537.2 & Oa4537.3 & Oa4537.4 & Oa4537.5 |
| Apolipoprotein E | APOE | Hs.515465 | Oa2757.1 & Oa2757.2 & Oa2757.3 |
| Lysyl-tRNA synthetase | KARS | Hs.3100 | Oa5596.1 & Oa5596.2 |
| Basigin | BSG | Hs.501293 | Oa4235.1 |
| Ribosomal protein L10 | RPL10 | Hs.401929 | Oa862.1 & Oa862.2 & Oa862.3 |
| Fc fragment of IgG, receptor, transporter, alpha | FCGRT | Hs.111903 | Oa1542.1 & Oa1542.2 |
| Cathepsin S | CTSS | Hs.181301 | Oa4958.1 & Oa4958.2 |
| Mortality factor 4 like 1 | MORF4L1 | Hs.374503 | Oa283.1 & Oa283.2 & Oa1092.1 |
| Glutathione peroxidase 3 | GPX3 | Hs.386793 | Oa4561.1 & Oa11520.1 |
| Endoglin | ENG | Hs.76753 | Oa3820.1 & Oa5229.1 |
| Tubulin, beta 2B | TUBB2 | Hs.300701 | Oa3697.2 & Oa3697.5 & Oa3697.8 |
| Ribosomal protein L10a | RPL10A | Hs.546269 | Oa3392.1 & Oa3392.2 |
| Ribosomal protein L6 | RPL6 | Hs.546283 | Oa1923.2 |
| Peptidylprolyl isomerase B (cyclophilin B) | PPIB | Hs.434937 | Oa1991.1 |
| Ribosomal protein S5 | RPS5 | Hs.378103 | Oa2009.1 & Oa17187.1 |
| HLA-A | Major histocompatibility complex, class I, A | Hs.181244 | Oa420.1 & Oa999.1 & Oa999.2 & Oa2302.1 & Oa2302.2 & Oa4619.1 & Oa4619.4 & Oa4619.7 & Oa4619.10 & Oa4688.1 & Oa5054.2 |
| CD74 antigen (invariant polypeptide of major histocompatibility complex, class II antigen-associated) | CD74 | Hs.436568 | Oa1531.1 & Oa1531.2 |
| Phosphoglycerate kinase 1 | PGK1 | Hs.78771 | Oa4344.1 |
| Nuclease-sensitive element-binding protein 1 | NSEP1 | Hs.512451 | Oa2431.1 & Oa2431.2 |
| Enolase 1, (alpha) | ENO1 | Hs.517145 | Oa3146.1 |
| Eukaryotic translation initiation factor 4A, isoform 1 | A1 | Hs.129673 | Oa1511.1 & Oa1511.2 & Oa5408.1 |
| Hepatoma-derived growth factor (high-mobility group protein 1-like) | HDGF | Hs.506748 | Oa3338.1 & Oa4571.1 |
| Annexin A2 | ANXA2 | Hs.511605 | Oa2002.2 & Oa2002.26 |
| Bone morphogenetic protein 1 | BMP1 | Hs.1274 | Oa3204.1 & Oa13162.1 & Oa14923.1 |
| Collagen, type I, alpha 2 | COL1A2 | Hs.489142 | Oa517.4 & Oa2002.1 & Oa2002.6 & Oa2002.7 & Oa2002.8 & Oa2002.9 & Oa2002.10 & Oa2002.11 & Oa2002.12 & Oa2002.13 & Oa2002.14 & Oa2002.15 & Oa2002.16 & Oa2002.17 & Oa2002.18 & Oa2002.19 & Oa2002.20 & Oa2002.22 & Oa2002.24 & Oa2002.29 |
| Collagen, type IV, alpha 2 | COL4A2 | Hs.508716 | Oa1632.1 & Oa4311.1 |
| Collagen, type VI, alpha 1 | COL6A1 | Hs.474053 | Oa5501.1 |
| Collagen, type VI, alpha 2 | COL6A2 | Hs.420269 | Oa2002.3 & Oa2002.25 |
| Cysteine and glycine-rich protein 1 | CSRP1 | Hs.108080 | Oa1190.1 & Oa2219.1 & Oa2219.2 & Oa2219.3 & Oa2219.4 & Oa2219.5 |
| Cathepsin D | CTSD | Hs.546248 | Oa2016.1 & Oa2016.2 & Oa2016.3 & Oa14044.1 |
| Glyceraldehyde-3-phosphate dehydrogenase | GAPDH | Hs.544577 | Oa1229.2 |
| Glutathione peroxidase 1 | GPX1 | Hs.76686 | Oa4732.1 |
| Glutathione peroxidase 4 | GPX4 | Hs.433951 | Oa3493.1 & Oa3493.2 |
| Glutathione peroxidase 7 | GPX7 | Hs.43728 | Oa2105.1 |
| Integrin-binding sialoprotein | IBSP | Hs.518726 | Oa476.1 & Oa476.2 & Oa476.3 |
| Inhibitor of DNA binding 1, dominant negative helix-loop-helix protein | ID1 | Hs.504609 | Oa598.1 & Oa2621.1 |
| Inhibitor of DNA binding 3, dominant negative helix-loop-helix protein | ID3 | Hs.76884 | Oa402.1 & Oa402.2 |
| Integrin, alpha 5 | ITGA5 | Hs.505654 | Oa431.1 & Oa2625.1 & Oa2725.1 & Oa4941.1 |
| Integrin, beta 1 | ITGB1 | Hs.429052 | Oa108.1 & Oa2887.1 |
| KDEL (Lys-Asp-Glu-Leu) endoplasmic reticulum protein retention receptor 2 | KDELR2 | Hs.520210 | Oa3649.1 & Oa4779.1 |
| KDEL (Lys-Asp-Glu-Leu) endoplasmic reticulum protein retention receptor 3 | KDELR3 | Hs.554798 | Oa460.1 |
| Low density lipoprotein-related protein 1 (alpha-2-macroglobulin receptor) | LRP1 | Hs.162757 | Oa2871.1 & Oa15045.1 & Oa16631.1 |
| Low density lipoprotein receptor-related protein 10 | LRP10 | Hs.525232 | Oa190.1 & Oa13504.1 |
| Lumican | LUM | Hs.406475 | Oa1923.1 & Oa1923.3 |
| Matrix metallopeptidase 2 (gelatinase A) | MMP2 | Hs.513617 | Oa5346.1 |
| Matrix metallopeptidase 9 (gelatinase B) | MMP9 | Hs.297413 | Oa2728.1 |
| Matrix metallopeptidase 13 (collagenase 3) | MMP13 | Hs.2936 | Oa811.1 & Oa5453.1 & Oa5453.2 |
| Matrix metallopeptidase 14 | MMP14 | Hs.2399 | Oa4537.1 & Oa4537.2 & Oa4537.3 & Oa4537.4 & Oa4537.5 |
| Matrix metallopeptidase 19 | MMP19 | Hs.154057 | Oa5670.1 |
| Heparan sulfate proteoglycan 2 (perlecan) | HSPG2 | Hs.555874 | Oa513.1 & Oa3349.1 |
| Retinoic acid receptor, alpha | RARA | Hs.535499 | Oa1624.1 & Oa4819.1 & Oa4819.2 & Oa13152.1 |
| Ribosomal protein L29 | RPL29 | Hs.425125 | Oa1866.1 |
| Ribosomal protein L41 | RPL41 | Hs.381172 | Oa1006.1 |
| S100 calcium binding protein A4 | S100A4 | Hs.81256 | Oa4271.1 |
| Serpin peptidase inhibitor, clade H (heat shock protein 47), member 1 | SERPINH1 | Hs.241579 | Oa4073.1 & Oa4073.2 & Oa4073.3 & Oa4073.4 & Oa4073.5 |
| Transgelin 2 | TAGLN2 | Hs.517168 | Oa646.5 & Oa646.29 & Oa646.30 |
| Transcription factor 4 | TCF4 | Hs.200285 | Oa4904.1 & Oa4904.2 & Oa4904.3 & Oa4904.4 |

## RT-PCR Primer

The
following table lists the RT-PCR primers developed for this study. The
following PCR program was used: 50�C : 2 min, 95�C : 10 min and then
40x (95�C : 15 sec, 60�C : 1 min) and finally for the registration of
the dissociation curve: 95�C : 15 sec, 60�C : 15 sec, 95�C : 15 sec

**Table 2.**

|  |  |  |  |
| --- | --- | --- | --- |
| Gene | Forward Primer | Reverse Primer | *Ovis aries* contig |
| ACP5 | TTGCCAGGACAGTGCAGATC | CTCCCAGGGACAGAACGAAG | Oa2093.1 |
| ACTA1 (Oa3284.3) | TTTCCCGTCTATCGTGGGAC | ATTCCCACCATCACTCCCTG | Oa3284.3 |
| ACTA1 (Oa3284.4) | TCGCAGACAGGATGCAGAAA | TCATTGTGCTAGGTGCCAGG | Oa3284.4 |
| ANXA2 | TGCAAGCTCAGTTTGGAGGG | CCCGTATGCACTTGGAGGTG | Oa2002.2 |
| APOE | CAGTTGGCTCTGCACCTCAG | TCAATGATTCTCGCTGGGC | Oa2757.1 |
| Basigin | ACAATGACGGCTCCAAAACG | TGGCACTGTGATTCAAGGCA | Oa4235.1 |
| BMP1 | AAGGCTGGCTTTGCTGTCAA | CCGAGAGCACTCATCCACCT | Oa3204.1 |
| COL1A1 | CCACCAGTCACCTGCGTACA | CACGTCTCGGTCATGGTACCT | Oa2002.1 |
| COL1A2 | CTCAGCTTTGTGGATACGCG | GCACGAAGTGACTGCAAGCA | Oa437.1 |
| COL3A1 | TTGGTCGCAAAGGTCATGC | CCTTAAAGCGGACTCCAGGA | Oa3746.1 |
| COL4A1 | CCTGATAGGAATCTGCCAGACTAAC | CAGCAGAGGCCAATGAAGC | Oa5752.1 |
| COL4A2 | CTCATCCGCACGCACATC | TCACAGGTTCTTCATGCACACC | Oa1632.1 |
| COL6A1 | GCCAGCAGAAACCACGACTC | GGTTTGCGGTCTGGGCTT | Oa5501.1 |
| COL6A2 | CAGAGAAGGCCGACTGCC | CCGATGTGTCCAGCACGA | Oa2002.3 |
| CSRP1 | TGCACGTGTCGCTAAGGACT | CAAACCAAAGGGAGCCAGAC | Oa2219.1 |
| CTSD | CTGAAATGCTGCTTGCTGGTC | CCGCCTCTCCATCAGACAGA | Oa2016.1 |
| CTSK | GCTTTGCTCTATACCCCGAGG | TCCATAGCTCCCATTGGGTG | Oa1508.1 |
| CTSS | CATGGATGGAAGGTGCCAGT | GAACACGTGGCAGCTCGATT | Oa4958.1 |
| Cystatin C | CCTTTGCGGTCAGCGAGTT | GGCTCTGGTAAGCGTCGTTG | Oa4147.1 |
| EEF1A1 | ACCACGACTGGCCATCTGAT | TGTTCTCTTGTCGATCCCGC | Oa3247.1 |
| EEF2 | ACCCAGTTGTCTCGTACCGG | AGAGCACGTTCGACTCCTCAC | Oa3405.1 |
| Endoglin | TCCTCCTTCGTCTGGACCAA | CCAGATTGCAGGAGAACGGT | Oa3820.1 |
| FCGRT | GGTCTGGGAAAGCCAGGTGT | AGGTCCGTGGTCTCTTTCTCC | Oa1542.1 |
| FTH | TGATGACTGGGAGAATGGGC | TCCAAGCACAGCGCACAT | Oa2373.1 |
| FTL | CCTTTTTTCCAGTCGCAACC | GATCGCGGACAGCTGAGTG | Oa753.1 |
| GFP (Green fluorescent protein) | TGTCGACACAATCTGCCCTTT | TGTGGTCACGCTTTTCGTTG | - |
| GNAI2 | TCAAGATGTTTGACGTGGGC | GGATCCACTTCTTCCGCTCA | Oa3850.1 |
| GPX1 | AATTGCCTGAAGTACGTCCGA | CATGAAATTGGGCTCGAACC | Oa4732.1 |
| GPX3 | TGAACCATTCGGTCTGGTCA | TTTCCAAATTGGTTGCAGGG | Oa4561.1 |
| GPX4 | GATGAAAGTCCAGCCCAAGG | CATTTGATGGCGTTTCCCAG | Oa3493.1 |
| GPX7 | CTGGAGAAGTACCGAGGCTCG | CTCGCTAGCCACATTCACCA | Oa2105.1 |
| HDGF | TGGGAGATCGAGAACAACCC | GGAGGACTGGTAGCCAGAAGC | Oa3338.1 |
| HMOX1 | AGGCCATCCCCTACACACAG | CCTGGAGTCGCTGAACATAGC | Oa1094.1 |
| HSPG2 | CCTCCTCTGGGTTGGGAAG | TGCCTTTTGCTCCACAGTTG | Oa513.1 |
| IBSP | GAATGGCCTGTGCTCTTTCC | CTAATTTGGCTCTTCGATGCAA | Oa476.1 |
| ID1 | TGGAGATCCTCCAGCACGTC | GCTCCAACTCCAGATCCCAG | Oa2621.1 |
| ID3 | CTGTGAACATGGGTGGCCTA | CAGCTGGTGCCCAGAGCTAG | Oa402.1 |
| ITGA5 | GTGGCCTTCGGTTCACAGTC | GGATGGTTTTCTTCGTGTCCC | Oa431.1 |
| ITGB1 | GTGTGGTTGCTGGGATTGTTC | TCCAAATCAGCAGCAATGCA | Oa2887.1 |
| ITM2C | GGAGAGCCGAGCGGTTGT | CAAAAGCCACTCGGTGCAG | Oa5278.1 |
| JUNB | GCAAGGGAGAAGCGACACC | CCTTTGCCCCTCTCTAGCG | Oa4766.1 |
| K-ALPHA-1 | TACCAGCCTCCCACTGTGGT | TCGCTGTACTTTGGCCAGGT | Oa4088.1 |
| KARS | GAGTCGTGTGACGGGCATTT | TCCCTCGCAGTCCCTGATTA | Oa5596.1 |
| KDELR2 | CTCAGTCTGCCCGCGTAAGT | CTGAGGACAGATGCTGCTGGT | Oa4779.1 |
| KDELR3 | GGAGGTCCAAGTGCTGCG | CGAAAAGGATTTGGCTCTTCC | Oa460.1 |
| LAMR1 | TTAACCTGCCAACCATTGCC | CGTAGCGCAGAGGAGAGTCC | Oa829.1 |
| LRP1 | CTTTGCTGCCCTTGTCTGGA | TTCCCTTCCTACCTCAGCCA | Oa2871.1 |
| LRP10 | TTCTGGGCAATCTGCGTTCT | GAGTCATGTCCTGGCGAAGG | Oa190.1 |
| Lumican | TCATCACCAAACTGTGCACCA | TGTGGGATAGCTTTCAGGGC | Oa1923.1 |
| MMP2 | GCTCTGCAAACAGGACATCG | TCCCCACGGATCTGAGAGAT | Oa5346.1 |
| MMP9 | CGGATTCCAGACCTTTGAGG | GTGATGTTGTGATGGTGCCAC | Oa2728.1 |
| MMP13 | AGCATGGCGACTTCTACCCA | CGTGAGCCAACAGACCAGAG | Oa811.1 |
| MMP14 | TCAAGGCCAATGTTCGAAGG | GCCATTTGAGTCCCTGGATG | Oa4537.1 |
| MMP19 | CTGCTCCCTCAGGTTCCCA | TTCAGAGTTTGTGCCCTCCG | Oa5670.1 |
| MORF4L1 | GGCGGCGGTGAATCTTTTAT | TTAGGCTTCGGGTCCTGCTT | Oa283.1 |
| Osteonectin | CTGTTGCCAGGCTCTAAGCC | GGGCAGTCTGGAGGTCTGC | Oa437.2 |
| PPIB | CAAGACAGATGGTCGGGACA | CAGTCTGCGATCGTCACGTC | Oa1991.1 |
| RARA | TGAAGGCCTGCTCTGGACC | AAGCGGTTCTGCGAGCTG | Oa4819.1 |
| RPL3 | TCACCGCCTTTCTTGGCTAC | CCTCCCTCACAATGTGGGTC | Oa691.1 |
| RPL6 | AGCCGAAATCCTGTCCTGGT | GGCTGATCGGGAATATCTGC | Oa1923.2 |
| RPL7 | AACCATGGAGGGTGCAGAAG | TCTGGCACAGCAGGAACCTT | Oa1986.1 |
| RPL7a | GAAGGTGGTCAACCCCCTGT | CCAATGCCAAAATTCTTGGG | Oa2982.1 |
| RPL9 | AGGGTGTTACACTGGGCTTCC | AGTGGGCATACACCGACCTC | Oa5542.1 |
| RPL10 | TGGTTTTCACATCCGAGTGC | TTGATGCGGATGACATGGAA | Oa862.1 |
| RPL10a | GGCACCGTCAGGCTTAAGTC | AACACACACGGAGAACTTGGG | Oa3392.1 |
| RPL17 | TTACAGAGCTCACGGTCGGA | ATGTGGCAGGGAGAGCTCAT | Oa4089.1 |
| RPL29 | CATGGCCAAGTCCAAGAACC | TGCCATTTTCGGGACTGGT | Oa1866.1 |
| RPL41 | TCGCCTTGACCACCTTTGAG | GGGACGGCTTTGATATGAGC | Oa1006.1 |
| RPLP0 | GCTTCATTGTGGGAGCAGACA | GGATCTGCTGCATCTGCTTG | Oa3428.1 |
| RPS2 | ACATGTCGGTCTGGGTGTCA | CGGATGGCAGTGGCTACTTC | Oa3320.1 |
| RPS3 | GCCGTGCAAATTTCCAAAAA | TTTGAAGATGCCATCAGCGA | Oa4417.1 |
| RPS4X | CCACTCGGCTTTCGAACATT | TCCATGGTTTGTTGCCTTTG | Oa729.1 |
| S100A4 | ATCGCCATGATGTGCAATGA | GGGCTGCTTATCAGGGAACC | Oa4271.1 |
| SERPINH1 | CAGCCTATACCAGGCCATGG | AACAGGATGTTCTCCACCGC | Oa4073.1 |
| SLC25A6 | TGAAGATCACCAAGTCCGACG | CGTTGAAGCCCTGGTACAGC | Oa5193.1 |
| TCF4 | GGCAAACGCAAGCAGAGTCT | TGGGTTGCCCATATCCATGT | Oa4904.1 |
| TPM2 | AGGAAAAGATGGAGCTGCAGG | CGATGTGCTTGGCCTCCTT | Oa1652.1 |
| Transgelin 2 | GCCCCAGTGAAGAAGATCCA | CTCCATCTGCTTGAAGGCCA | Oa646.5 |
| UBC | GTGGCTGTTAATTCTTCAGTCTTCTG | CGATGCCATCACTGAGCATT | Oa4596.1 |

## RT-PCR Results

**Table 3.**

|  |  |  |  |  |
| --- | --- | --- | --- | --- |
| Gene | 7d | 10d | 14d | 42d |
| ACP5 | 1.000 +/- 0.051 | 4.073 +/- 0.203 | 2.950 +/- 0.111 | 1.806 +/- 0.03 |
| ACTA1 (Oa3284.3) | 1.000 +/- 0.03 | 0.597 +/- 0.018 | 0.495 +/- 0.022 | 0.211 +/- 0.007 |
| ACTA1 (Oa3284.4) | 1.000 +/- 0.037 | 0.641 +/- 0.01 | 0.353 +/- 0.053 | 0.149 +/- 0.004 |
| ANXA2 | 1.000 +/- 0.021 | 0.856 +/- 0.029 | 0.700 +/- 0.023 | 0.480 +/- 0.022 |
| APOE | 1.000 +/- 0.034 | 0.741 +/- 0.128 | 0.852 +/- 0.012 | 0.507 +/- 0.017 |
| Basigin | 1.000 +/- 0.032 | 0.771 +/- 0.022 | 0.573 +/- 0.009 | 0.339 +/- 0.054 |
| BMP1 | 1.000 +/- 0.031 | 0.923 +/- 0.061 | 0.591 +/- 0.014 | 0.210 +/- 0.009 |
| COL1A1 | 1.000 +/- 0.034 | 1.361 +/- 0.052 | 1.150 +/- 0.054 | 0.435 +/- 0.015 |
| COL1A2 | 1.000 +/- 0.036 | 1.523 +/- 0.063 | 1.225 +/- 0.049 | 0.495 +/- 0.038 |
| COL3A1 | 1.000 +/- 0.035 | 0.765 +/- 0.027 | 0.514 +/- 0.013 | 0.367 +/- 0.005 |
| COL4A1 | 1.000 +/- 0.038 | 0.535 +/- 0.10 | 0.696 +/- 0.009 | 0.295 +/- 0.031 |
| COL4A2 | 1.000 +/- 0.026 | 0.648 +/- 0.031 | 0.695 +/- 0.006 | 0.326 +/- 0.013 |
| COL6A1 | 1.000 +/- 0.039 | 1.151 +/- 0.21 | 0.971 +/- 0.021 | 0.530 +/- 0.02 |
| COL6A2 | 1.000 +/- 0.032 | 0.843 +/- 0.044 | 0.707 +/- 0.036 | 0.315 +/- 0.011 |
| CSRP1 | 1.000 +/- 0.069 | 0.677 +/- 0.026 | 0.530 +/- 0.033 | 0.292 +/- 0.007 |
| CTSD | 1.000 +/- 0.034 | 0.474 +/- 0.016 | 0.731 +/- 0.022 | 0.095 +/- 0.005 |
| CTSK | 1.000 +/- 0.040 | 2.912 +/- 0.134 | 2.576 +/- 0.087 | 2.561 +/- 0.136 |
| CTSS (Cathepsin S) | 1.000 +/- 0.04 | 0.695 +/- 0.016 | 0.710 +/- 0.038 | 0.139 +/- 0.005 |
| Cystatin C | 1.000 +/- 0.063 | 1.130 +/- 0.057 | 1.267 +/- 0.096 | 2.572 +/- 0.062 |
| EE1A1 | 1.000 +/- 0.039 | 0.842 +/- 0.046 | 0.638 +/- 0.01 | 0.519 +/- 0.006 |
| EEF2 | 1.000 +/- 0.04 | 0.806 +/- 0.035 | 0.490 +/- 0.012 | 0.322 +/- 0.011 |
| Endoglin | 1.000 +/- 0.033 | 0.719 +/- 0.028 | 0.688 +/- 0.031 | 0.410 +/- 0.016 |
| FCGRT | 1.000 +/- 0.079 | 0.830 +/- 0.029 | 0.790 +/- 0.017 | 0.315 +/- 0.014 |
| FTH | 1.000 +/- 0.07 | 0.747 +/- 0.026 | 0.000 +/- 0.00 | 0.180 +/- 0.006 |
| FTL | 1.000 +/- 0.087 | 0.537 +/- 0.026 | 0.515 +/- 0.012 | 0.170 +/- 0.003 |
| GAPDH | 1.000 +/- 0.062 | 0.681 +/- 0.011 | 0.436 +/- 0.011 | 0.417 +/- 0.005 |
| GNAI2 | 1.000 +/- 0.04 | 0.740 +/- 0.056 | 0.506 +/- 0.002 | 0.210 +/- 0.004 |
| GPX1 | 1.000 +/- 0.012 | 0.802 +/- 0.024 | 0.618 +/- 0.016 | 0.208 +/- 0.018 |
| GPX3 | 1.000 +/- 0.02 | 0.944 +/- 0.011 | 0.502 +/- 0.016 | 0.615 +/- 0.014 |
| GPX4 | 1.000 +/- 0.036 | 0.851 +/- 0.031 | 0.619 +/- 0.02 | 0.301 +/- 0.016 |
| GPX7 | 1.000 +/- 0.008 | 1.089 +/- 0.029 | 0.732 +/- 0.012 | 0.441 +/- 0.02 |
| HDGF | 1.000 +/- 0.047 | 0.639 +/- 0.111 | 0.662 +/- 0.029 | 0.337 +/- 0.024 |
| HMOX1 | 1.000 +/- 0.027 | 0.350 +/- 0.014 | 0.327 +/- 0.04 | 0.019 +/- 0.001 |
| IBSP | 0.042 +/- 0.005 | 1.000 +/- 0.049 | 0.661 +/- 0.019 | 0.774 +/- 0.021 |
| ID1 | 1.000 +/- 0.038 | 1.149 +/- 0.034 | 0.877 +/- 0.065 | 0.474 +/- 0.022 |
| ID3 | 1.000 +/- 0.117 | 1.151 +/- 0.024 | 0.806 +/- 0.035 | 0.682 +/- 0.06 |
| ITGA5 | 1.000 +/- 0.042 | 0.702 +/- 0.02 | 0.401 +/- 0.039 | 0.191 +/- 0.009 |
| ITGB1 | 1.000 +/- 0.041 | 0.735 +/- 0.041 | 0.509 +/- 0.025 | 0.293 +/- 0.01 |
| ITM2C | 1.000 +/- 0.053 | 1.143 +/- 0.029 | 0.939 +/- 0.056 | 0.338 +/- 0.022 |
| JUNB | 1.000 +/- 0.02 | 0.893 +/- 0.039 | 0.559 +/- 0.087 | 0.297 +/- 0.009 |
| K-ALPHA-1 | 1.000 +/- 0.028 | 0.680 +/- 0.018 | 0.323 +/- 0.01 | 0.188 +/- 0.005 |
| KARS | 1.000 +/- 0.06 | 0.762 +/- 0.058 | 0.615 +/- 0.083 | 0.556 +/- 0.035 |
| KDELR2 | 1.000 +/- 0.036 | 0.912 +/- 0.045 | 0.488 +/- 0.027 | 0.314 +/- 0.015 |
| KDELR3 | 1.000 +/- 0.037 | 0.951 +/- 0.019 | 0.625 +/- 0.013 | 0.299 +/- 0.036 |
| LAMR1 | 1.000 +/- 0.023 | 0.799 +/- 0.039 | 0.604 +/- 0.075 | 0.310 +/- 0.01 |
| LRP1 | 1.000 +/- 0.043 | 0.901 +/- 0.033 | 0.674 +/- 0.081 | 0.352 +/- 0.016 |
| LRP10 | 1.000 +/- 0.034 | 0.839 +/- 0.083 | 0.716 +/- 0.042 | 0.421 +/- 0.031 |
| Lumican | 1.000 +/- 0.012 | 1.082 +/- 0.017 | 0.740 +/- 0.027 | 0.501 +/- 0.007 |
| MMP2 | 1.000 +/- 0.045 | 1.182 +/- 0.049 | 0.980 +/- 0.043 | 0.538 +/- 0.015 |
| MMP9 | 1.000 +/- 0.112 | 5.990 +/- 0.212 | 3.036 +/- 0.076 | 1.881 +/- 0.507 |
| MMP13 | 1.000 +/- 0.042 | 6.098 +/- 0.248 | 4.470 +/- 0.111 | 5.501 +/- 0.162 |
| MMP14 | 1.000 +/- 0.035 | 1.265 +/- 0.041 | 0.993 +/- 0.028 | 0.368 +/- 0.009 |
| MMP19 | 1.000 +/- 0.039 | 0.859 +/- 0.041 | 0.806 +/- 0.109 | 0.210 +/- 0.008 |
| MORF4L1 | 1.000 +/- 0.034 | 0.775 +/- 0.025 | 0.684 +/- 0.082 | 0.496 +/- 0.006 |
| Osteonectin | 1.000 +/- 0.033 | 0.844 +/- 0.068 | 0.750 +/- 0.005 | 0.567 +/- 0.017 |
| Perlecan (HSPG2) | 1.000 +/- 0.030 | 0.691 +/- 0.013 | 0.706 +/- 0.015 | 0.363 +/- 0.012 |
| PPIB | 1.000 +/- 0.022 | 0.884 +/- 0.04 | 0.751 +/- 0.111 | 0.268 +/- 0.008 |
| RARA | 1.000 +/- 0.067 | 0.729 +/- 0.043 | 0.623 +/- 0.015 | 0.447 +/- 0.024 |
| RPL3 | 1.000 +/- 0.046 | 0.807 +/- 0.014 | 0.701 +/- 0.025 | 0.439 +/- 0.004 |
| RPL6 | 1.000 +/- 0.014 | 0.851 +/- 0.03 | 0.632 +/- 0.075 | 0.453 +/- 0.005 |
| RPL7 | 1.000 +/- 0.021 | 0.801 +/- 0.036 | 0.539 +/- 0.065 | 0.412 +/- 0.009 |
| RPL7a | 1.000 +/- 0.061 | 0.872 +/- 0.032 | 0.677 +/- 0.081 | 0.461 +/- 0.029 |
| RPL9 | 1.000 +/- 0.027 | 0.810 +/- 0.028 | 0.623 +/- 0.074 | 0.456 +/- 0.01 |
| RPL10 | 1.000 +/- 0.024 | 0.689 +/- 0.027 | 0.461 +/- 0.056 | 0.425 +/- 0.008 |
| RPL10a | 1.000 +/- 0.028 | 0.810 +/- 0.028 | 0.546 +/- 0.021 | 0.451 +/- 0.018 |
| RPL17 | 1.000 +/- 0.029 | 0.800 +/- 0.024 | 0.635 +/- 0.015 | 0.470 +/- 0.011 |
| RPL29 | 1.000 +/- 0.014 | 0.815 +/- 0.008 | 0.549 +/- 0.016 | 0.436 +/- 0.012 |
| RPL41 | 1.000 +/- 0.015 | 0.712 +/- 0.015 | 0.092 +/- 0.003 | 0.423 +/- 0.023 |
| RPLP0 | 1.000 +/- 0.036 | 0.763 +/- 0.022 | 0.654 +/- 0.014 | 0.383 +/- 0.011 |
| RPS2 | 1.000 +/- 0.012 | 0.817 +/- 0.008 | 0.525 +/- 0.013 | 0.360 +/- 0.008 |
| RPS3 | 1.000 +/- 0.036 | 0.751 +/- 0.025 | 0.569 +/- 0.038 | 0.332 +/- 0.034 |
| RPS4X | 1.000 +/- 0.05 | 0.795 +/- 0.044 | 0.523 +/- 0.018 | 0.426 +/- 0.016 |
| S100A4 | 1.000 +/- 0.027 | 0.879 +/- 0.035 | 0.560 +/- 0.011 | 0.574 +/- 0.02 |
| SERPINH1 | 1.000 +/- 0.011 | 0.801 +/- 0.01 | 0.595 +/- 0.019 | 0.153 +/- 0.003 |
| SLC25A6 | 1.000 +/- 0.039 | 0.796 +/- 0.027 | 0.549 +/- 0.037 | 0.270 +/- 0.011 |
| TCF4 | 1.000 +/- 0.018 | 0.693 +/- 0.041 | 0.588 +/- 0.011 | 0.517 +/- 0.025 |
| TPM2 | 1.000 +/- 0.066 | 0.941 +/- 0.066 | 0.557 +/- 0.041 | 4.109 +/- 0.163 |
| Transgelin 2 | 1.000 +/- 0.038 | 0.697 +/- 0.018 | 0.561 +/- 0.017 | 0.253 +/- 0.004 |
| UBC | 1.000 +/- 0.034 | 0.942 +/- 0.009 | 0.846 +/- 0.019 | 0.471 +/- 0.035 |
